# Patient-reported understanding and dentist-reported management of periodontal diseases - a survey: do you know what gum disease is?

**DOI:** 10.1038/s41415-023-6055-7

**Published:** 2023-07-28

**Authors:** Haajarah Rana, Barbara Warnes, Maria Davies, Nicola X. West

**Affiliations:** Clinical Trials Group, Bristol Dental School, University of Bristol, Lower Maudlin Street, Bristol, BS1 2LY, United Kingdom

## Abstract

**Introduction **Mild-to-moderate gingivitis is treatable by effective toothbrushing with appropriate over-the-counter oral health care products; however, rates remain high.

**Aim** To determine patient knowledge of gingivitis and dentists' views on management.

**Methods** Surveys were completed by dentists and dental hospital patients.

**Results** In total, 224 patients and 50 dentists participated. Clinical health, gingivitis, or periodontitis was detected in 2%, 33% and 56% of patients, respectively; 32% reported never suffering gingival bleeding. Moreover, 74% of patients reported gingival health as very important but only 53.7% with gingivitis occasionally/often were moderately-extremely worried about their symptoms. More than 50% of patients knew gingivitis causes poor oral health but <20% knew it elevated risks of other systemic conditions. Patients thought education on risks associated with poor oral health and product recommendations were most likely, and daily reminders least likely, to improve compliance with oral health advice (OHA). Also, 40% of dentists thought their patients were relatively unaware of the importance of gingival health, 76.9% of their patient-base had gingivitis, and 96% give OHA to these patients but only 30% thought this effected improvement. The most useful tools for improving oral health were better patient knowledge of the consequences and one-to-one instruction.

**Conclusion** Patients struggle to attain oral health following OHA. Education about gingivitis-associated risks might improve OHA compliance.

## Key points

- Most patients were not aware of their periodontal diseases.
- Dentists were generally not concerned about gingivitis as this condition is reversible; however, they acknowledge with their patients that compliance with oral hygiene recommendations is often short-lived.
- Patients and dentists thought that awareness of risks of non-compliance with oral hygiene advice might improve compliance.
- Patients were largely unaware of the increased risks associated with non-oral diseases, such as diabetes and infective endocarditis.

## Introduction

Periodontal diseases are very common worldwide, with severe periodontitis being the sixth most prevalent condition globally, affecting 11.2% of the population.^1^ Untreated periodontitis can lead to tooth loss and associated negative effects on quality of life, arising from functional problems encountered when eating and psychological problems caused by having missing teeth.^2^ Periodontitis is also associated with an increasing number of non-oral health problems, including diabetes, cardiovascular disease and Alzheimer's disease.^3^ The precursor to periodontitis is gingivitis, which, if untreated, will progress to periodontitis in most individuals.^4^^,^^5^ Unlike periodontitis, gingivitis is reversible; therefore, oral hygiene measures should target gingivitis for the primary prevention of periodontitis and secondary prevention of recurrent periodontitis.^6^

Prevalence rates for gingivitis are high. In Europe, approximately 75% of 15-19-year-olds had bleeding on probing with or without calculus, a prevalence that decreased with age as the prevalence of periodontitis increased,^7^ and in a UK study, 76% of dental attenders aged 18-92 had bleeding on probing.^8^ These high rates of gingivitis highlight a need for better communication to patients/the public about how they should be caring for their teeth and gums to achieve periodontal health/stability, and the risks associated with not following this guidance.

To determine how to improve awareness of the importance of gingival health and enable patients to make improvements to their oral hygiene, it is necessary to know what information they are being given, what they retain, and what they believe might help them make changes that result in long-term periodontal health.

This study aimed to determine patient understanding of gingivitis, their oral hygiene practices and what might help them follow oral hygiene advice. Dentist views on the gravity of gingivitis, how they treat it, and the advice/sources of information they believe are most useful for patients were also captured.

## Methods

This was a two-part questionnaire-based survey that was deemed to be a service evaluation, rather than research, by the trust from which the patient participants were recruited (University Hospitals Bristol NHS Foundation Trust) hence, ethical approval was not required, and service evaluation approval was obtained from the trust. The non-validated questionnaire was developed in line with the requirements of the trust's approval board, and the service evaluation 'Do you know what gum disease is? A survey' was approved by the trust's questionnaire, interview and survey (QIS) panel in February 2018.

Patients attending Bristol Dental Hospital (BDH) for routine appointments were provided with the QIS-approved information sheet. Study staff went through the information sheet with patients who expressed an interest in participating, answered any questions they had, and provided them with the questionnaire regarding their understanding, attitudes and behaviours around gingivitis. Consent was deemed obtained if participants completed the fully anonymous questionnaire, placed it in a sealed envelope and returned the sealed envelope to their treating clinician. The patient's Basic Periodontal Examination (BPE) score and subsequent periodontal diagnosis were recorded on the outside of the envelope. No patient identifiers were collected. As this study was started before the publication of the new classification scheme for periodontal diseases,^9^ staging and grading were not conducted.

Qualified dentists working at BDH were emailed a QIS-approved information sheet. Dentists who agreed to take part completed a questionnaire about the knowledge they perceived their patients had about gingivitis, methods that could be used to improve patient knowledge, their own perceptions of the seriousness of gingivitis and how they routinely treat it. Consent was deemed obtained if dentists completed the fully anonymous questionnaire and deposited it in a drop box on clinic. No dentist identifiers were captured.

## Results

The study was carried out from March 2018 to February 2019 and recruited 224 patient participants: 46% were men, 50% were women and 4% gave no response. Participant number increased with age-group, with 29% aged 65+.

Clinically, only 2% of patients had gingival health, 33% had gingivitis and 56% had periodontitis. However, 32% of respondents reported never suffering gingival bleeding after brushing (BAB) and 45% indicated they never suffered from gingival inflammation. No periodontal disease diagnosis could be linked to the questionnaire responses for 9% of participants. Evidence of periodontitis was more common in individuals aged 35+ and 19% reported family history of periodontitis, which was twice as common in participants with periodontitis than gingivitis (23% vs 10%).

**Fig. 1:**
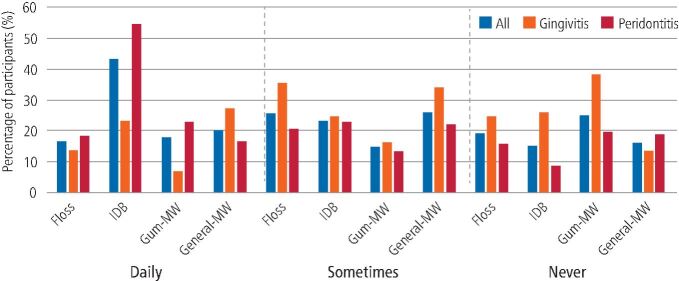
Percentage of participants that used additional cleaning aids and the frequency of use

Most participants (70%) brushed their teeth twice daily. Compared to those with gingivitis, those with periodontitis were more likely to brush only once (18% vs 4%) or less than once a day (2% vs 0%). Overall, more participants used a power brush than a manual brush (47% vs 32%), with the remainder using both brush types. Manual brush use was more common in those with gingivitis (43% vs 21%). Use of additional cleaning aids is shown in Figure 1. Daily use of interdental brushes (IDBs) was most commonly reported (44%), compared to 17-20% daily use of floss or mouthwash. IDBs and gingivitis-specific mouthwashes were twice as likely to be used daily by those with periodontitis compared to gingivitis.

The relative importance of fresh breath, healthy gingivae, healthy teeth and a clean tongue were rated as very important by 74%, 74%, 73%, and 57% of participants, respectively, and almost all of those who did not rate these as very important rated them as important. White teeth were considered less important, with only 30% rating them very important, and 4% indicating that they were not at all important. Responses were similar for those with gingivitis and periodontitis.

Participant concerns about their own oral health (Table 1) indicated that 12-15% of participants were often aware of at least one of the following issues: BAB, gingival inflammation (GI) or tender gums (TG), and 44-54% were aware of these occasionally. Concern about symptoms and recognition that they could lead to more serious periodontal health problems increased with the number of symptoms experienced often. Those with periodontitis were more likely to suffer symptoms and be very or extremely worried about these than those with gingivitis. The most common actions taken by those who reported BAB, GI or TG occasionally or often were to spend a bit more time and effort brushing (54%), use a gingivitis mouthwash (32%), talk to their dentist at their check-up (32%) and buy a new toothbrush (20%).

**Table 1:** Frequency of gingival conditions indicative of poor oral health and how worried participants were about them

Symptom	Frequency	All (%)*	Gingivitis (%)**	Periodontitis (%)?
Bleeding after brushing	Often	14.9	10.3	16
	Occasionally	53.4	48.5	56.3
	Never	31.7	41.2	27.7
Gingival inflammation	Often	11.5	4.7	12.3
	Occasionally	43.5	31.3	52.8
	Never	45	64.1	34.9
Tender gums	Often	13.2	5.9	14.8
	Occasionally	52.2	48.5	56.5
	Never	34.6	45.6	28.7
**Answered only by those who said often/occasionally**		**All** **(%, n = 175)**	**Gingivitis** **(%, n = 61)**	**Periodontitis** **(%, n = 98)**
Thinking about your symptoms, how worried are you by them?	Not at all	3.4	6.9	2
	Not especially	20	29.3	12.1
	Slightly	22.9	24.1	22.2
	Moderately	30.3	31	32.3
	Very	15.4	6.9	20.2
	Extremely	8	1.7	11.1
Key: * = BAB: n = 208; GI: n = 191; TG: n = 205. ** = BAB: n = 69; GI: n = 66; TG: n = 71. † = BAB: n = 118; GI: n = 105; TG: n = 114.				

Most participants were aware that poor gingival health is linked to oral conditions, with 60%, 55%, 54% and 52% reporting awareness regarding tooth loss, sensitive teeth, wobbly teeth and bad breath, respectively. Fewer participants were aware of the links between poor gingival health and non-oral conditions, with 16%, 15%, 6% and 5% aware of associations with diabetes, cardiovascular disease, dementia and irritable bowel syndrome, respectively. Responses of those with gingivitis and periodontitis were similar.

Most participants had received advice regarding brushing technique and interdental cleaning from their dentist/hygienist, and approximately one-third had been advised regarding the best toothbrush, or to use a mouthwash (Table 2). Overall, participants with periodontitis were slightly more likely to have been given oral hygiene advice (OHA) than those with gingivitis. OHA was followed by 59% of participants, but 40% said that after starting off well, they struggled to continue following their OHA. Understanding what might happen if OHA was not followed was most commonly selected as what might improve compliance, followed by recommendations for toothpaste or mouthwash, and being shown brushing/flossing. Daily reminders about OHA were indicated to be least likely to improve adherence to advice (Table 2).

**Table 2:** Advice received by participants, whether it was followed, and what might help adherence to advice

Oral health advice received in the past (all participants)*	All (%) (n = 222)	Gingivitis (%) (n = 76)	Periodontitis (%) (n = 123)
None	9.3	9.3	7.3
Brushing technique	74.1	65.3	78
Specific toothbrush	32.9	28	35.8
Interdental floss/brush	73.6	66.7	78.9
Mouthwash	29.6	66.7	33.3
Specific toothpaste	18.1	22.7	15.4
Dietary	13	24	11.4
Smoking cessation	18.5	10.7	21.1
**Was advice followed? (All participants who had received advice)**	**All (%)** **(n = 189)**	**Gingivitis (%)** **(n = 63)**	**Periodontitis (%)** **(n = 112)**
Always follow advice	58.7	55.7	60.2
Follow to start with, then struggle	39.7	44.3	37.2
Have difficulty following advice	1.6	0	2.7
What might help you follow advice (all participants)*	**All (%)** **(n = 210)**	**Gingivitis (%)** **(n = 74)**	**Periodontitis (%)** **(n = 116)**
Understanding what will happen if I don't follow the advice	57.1	52.1	59
Being shown how to brush and floss correctly	41.9	45.2	40.2
Being shown pictures by the dentist to help explain gum health	24.8	16.4	30.8
Toothpaste/mouthwash recommendation	43.8	47.9	43.6
Toothpaste/mouthwash sample from the dentist	32.4	34.2	31.6
A leaflet about how to improve gum health	35.7	26	42.7
Daily reminders (for example, app/text/note in bathroom)	11	9.6	11.1
Buying a power brush	21.9	24.7	19.7
A brush app that encourages two-minute brushing	15.7	17.8	15.4
"Key: * = All participants who answered the question"			

In the dentist survey, on average (across the 50 dentists surveyed), dentists reported that 76.9% of their patient-bases had gingivitis or periodontitis. Most dentists (68%) indicated having no or only slight concern if a patient had an overall score of BPE1; the reason they gave for this was because gingivitis is readily treatable. Dentists who indicated greater concern were more likely to highlight that gingivitis is active disease and carries a risk of progression. OHA was the standard approach for treating patients with gingivitis, with some dentists adding that they would include information about inflammation/progression risk when giving OHA.

Almost all dentists (92%) associated bleeding on probing with gingivitis and felt that this level of gingivitis mattered due to the progression risk. Only 15% of dentists thought that patients would think gingivitis was one of the most important components of oral health, 40% thought they would be relatively unaware of its importance, and the remainder indicated that patients might be aware but would rate it less important than caries. Only 6% of dentists thought that patients would know what to do if they had symptoms of gingivitis. Most dentists indicated that patients with symptoms of gingivitis should book a dental appointment, while 29% thought that the patient should wait and bring it up at their next check-up.

All dentists thought dentists or hygienists were a good source of information regarding periodontal health and 22% of dentists felt that a doctor was an inappropriate source of information regarding periodontal health. Dentists most commonly recommended use of IDBs (68%) or a power toothbrush (50%) to help patients with gingivitis, with a gingivitis-specific serum/gel and essential oils mouthwash most commonly reported as being inappropriate (56% and 60% of dentists, respectively). The most useful tools for improving oral health outcomes in patients were identified as better patient understanding of the consequences of poor oral health, and one-to-one demonstrations, with 90% and 84% of dentists indicating these were important. Specially formulated anti-gingivitis toothpaste and brushing apps were generally reported as not useful.

Only 30% of dentists thought their OHA triggered long-term changes in oral health practices. More commonly, it was felt that OHA was only effective in the short-term. Most dentists thought that further training on periodontal management, a short training course on using behaviour change methods, effective oral health care products, and visual aids and tools that can act as reminders to practice oral health, would all be beneficial for their practice.

## Discussion

This study explored periodontal diseases from various perspectives, including how serious both the public and dentists think it is, and tools to help patients comply with OHA provided to manage it/improve periodontal health.

Similar numbers of female and male participants took part in the patient survey, and all age groups were well-represented, although 65+ was the largest participant group, reflecting the demographics of clinic patients.

Only 2% of patient participants had healthy gingivae as determined clinically, while 56% had evidence of periodontitis. By contrast, bleeding on probing was reported in 53% and 76% of participants in studies by the UK Government^10^ and Midwood *et al.*^8^, respectively, and periodontal pocketing >4 in 28%^8^ and 45% of adults.^11^ The higher levels of gingivitis and periodontitis observed in the present study may reflect the relatively large number of older participants, as periodontal disease increases with age,^12^ although there was a similar age demographic in the study by UK Government.^10^

In the present study, nearly one-fifth of participants reported a family history of periodontal diseases, and those with periodontitis were twice as likely to report this. While genetic risk factors for periodontitis are known,^13^ and prevalence can be high in some families,^14^ literature regarding the prevalence of periodontitis with family history in the general population is limited. In a periodontal clinic, 37% of patients had a family history of periodontitis,^15^ which is higher than the figure obtained in the present study (23%), but may reflect the fact that participants enrolled in the current study were attending all-adult clinics.

In the present study, almost 50% of participants used a power brush, and power brush use was more common in those with periodontitis. Similarly, IDBs were used daily by a large proportion of participants (>40%) and by twice as many patients with periodontitis than with gingivitis, likely because IDBs are recommended to patients with periodontitis when they have gingival inflammation.^6^ The figures for power brush and IDB use are both much higher than the 26% and 6% reported in the 2009 Adult Dental Health Survey (ADHS),^16^ but ADHS participants used more floss and wood sticks, and the lack of power brush and IDB use could be due to their availability at that time. That both power brushes and IDBs were used more by those with periodontitis than gingivitis in the current study may be a result of them having been given more OHA specific to gingival health due to their periodontal disease, this being the correct treatment in a teaching hospital.

In the present study, >50% dentists indicated that IDBs and/or power brushes should be used by patients with gingivitis. Systematic reviews have confirmed the benefit of the use of power brushes as compared to a manual toothbrush for those with gingivitis,^17^ but not for patients in supportive periodontal care.^18^ IDBs have been demonstrated to be a priority for those with periodontitis, and there is weak evidence for a benefit for improving periodontal health in those with gingivitis, as long as the patient uses the appropriate size.^18^^,^^19^^,^^20^ Thus, most patient participants in the present study were using appropriate oral hygiene aids and most dentist participants are strongly recommending them.

Interestingly, while dentists in the present study thought that their patients would be less aware of gingivitis than caries, almost three-quarters of patient participants rated healthy gums and healthy teeth equally as very important, suggesting a good awareness of the importance of gingival health. However, the number of patients that reported daily symptoms of gingivitis was much lower than the number with periodontal diseases, suggesting patients did not recognise their own symptoms. A recent UK study also demonstrated that regular dental attenders with overall good oral health reported oral health conditions with less frequency than these conditions were detected clinically.^6^

Generally, neither patient nor dentist participants in the present study were overly worried about gingivitis. Dentists indicated their lack of concern was because gingivitis is reversible with the correct treatment,^21^ while patients weren't worried if they were only aware of suffering from symptoms occasionally or not at all. The patients who did indicate a level of anxiety were those who reported awareness of one or more symptoms of gingivitis often, and this group were also more knowledgeable about the risks associated with these symptoms. The dentists who expressed more concern about gingivitis mainly cited the risk of progression to more serious disease as the reason for their answer. While not all those with gingivitis go on to get periodontitis,^4^ it is a continuum of the same inflammatory disease,^22^ and the management of gingivitis is key in the prevention strategy for periodontitis.^6^ Indeed, the role of home care by patients is of paramount importance to prevent gingivitis and periodontitis. Economic analysis shows that both eliminating gingivitis using home care prevention techniques (for example, toothbrushing, IDBs) and increasing the diagnosis rate of periodontitis to 90%, with all patients diagnosed being managed, will have a positive return on investment in the UK.^23^

Dentists in the present study reported giving OHA to patients with gingivitis but were not asked to specify what this was. Most patient participants reported having received advice regarding toothbrushing and IDB technique, in line with what the dentists indicated they would recommend, advice that is supported by systematic reviews.^6^^,^^18^ However, 40% of patients reported struggling to follow the advice over a prolonged time, which aligned with the dentists' perceptions that patients start off well, but most are unable to make changes that last. Most patient participants thought that understanding the risks of not following advice, being shown the correct oral hygiene techniques, or being recommended a mouthwash, would improve their compliance, which aligned with dentist opinion regarding the most important things to support patients to improve gingival health. In addition, more than half of the dentists also indicated use of behaviour change methods, purchase of a power toothbrush, or regular reminders about oral hygiene as good support tools. However, the use of cards indicating patient risk with images of patients' dental plaque and the provision of verbal advice only yielded small improvements in patient-reported toothbrushing, with no differences between the groups, in a recent study.^24^

It is recognised that changing behaviours to maintain improved oral hygiene over prolonged periods is difficult, which is why, despite receiving OHA, many regular dental attenders still have gingivitis or periodontitis.^8^^,^^10^ Behaviour-change techniques have been shown to be successful for smoking cessation^25^ and literature indicates that interventions that include goal setting, self-monitoring and planning are effective for improving oral hygiene behaviours.^26^ In a recent study, an intervention that included goal setting and planning improved gingival health in those that received it as compared to those who received standard OHA.^27^ Most dentist participants in the present study indicated behaviour change was an area in which they would like more training, but they also indicated they would like training in other areas of OHA.

## Conclusion

Overall, this study demonstrated that patients attending a UK dental hospital for general restorative dental treatment had a higher prevalence of gingivitis and periodontitis than has been described for the population in recent surveys, but that they were relatively knowledgeable about periodontal diseases and were using the correct oral hygiene tools to try to improve their oral health, particularly those with periodontitis. Dentists were providing appropriate advice to their patients with gingivitis, but both the dentists and patients in the study recognised that they were struggling to improve oral hygiene in the long-term, which is essential for oral health stability. Inclusion of an overview of the oral and non-oral risks associated with gingivitis may improve oral health outcomes, and more training in the use of behaviour change techniques might improve the delivery of oral health interventions and should be a focus for continuing professional development.
